# Climate displaces deposition as dominant driver of dissolved organic carbon concentrations in historically acidified lakes

**DOI:** 10.1007/s10533-024-01193-5

**Published:** 2024-12-21

**Authors:** Allison M. Herreid, Hannah M. Fazekas, Sarah J. Nelson, Adam S. Wymore, Desneiges Murray, Ruth K. Varner, William H. McDowell

**Affiliations:** 1 Department of Natural Resources and the Environment, University of New Hampshire, 56 College Road, Durham, NH USA; 2 Present Address: U.S. Department of Agriculture–Agricultural Research Service, Soil and Water Management Research, University of Minnesota, 1991 Upper Buford Circle, St. Paul, MN USA; 3 Present Address: Biology Department, Saginaw Valley State University, University Center, MI USA; 4 Research Department, Appalachian Mountain Club, 361 Route 16, Gorham, NH 03581 USA; 5 Department of Earth Sciences and the Institute for the Study of Earth, Oceans, and Space, University of New Hampshire, 8 College Road, Durham, NH USA; 6 Institute of Environment, Florida International University, 11200 SW 8th Street, OE148, Miami, FL 33199 USA

**Keywords:** Climate, Dissolved organic carbon, Lake, Acidic deposition, Winter climate change, Browning

## Abstract

Climate and atmospheric deposition interact with watershed properties to drive dissolved organic carbon (DOC) concentrations in lakes. Because drivers of DOC concentration are inter-related and interact, it is challenging to assign a single dominant driver to changes in lake DOC concentration across spatiotemporal scales. Leveraging forty years of data across sixteen lakes, we used structural equation modeling to show that the impact of climate, as moderated by watershed characteristics, has become more dominant in recent decades, superseding the influence of sulfate deposition that was observed in the 1980s. An increased percentage of winter precipitation falling as rain was associated with elevated spring DOC concentrations, suggesting a mechanistic coupling between climate and DOC increases that will persist in coming decades as northern latitudes continue to warm. Drainage lakes situated in watersheds with fine-textured, deep soils and larger watershed areas exhibit greater variability in lake DOC concentrations compared to both seepage and drainage lakes with coarser, shallower soils, and smaller watershed areas. Capturing the spatial variability in interactions between climatic impacts and localized watershed characteristics is crucial for forecasting lentic carbon and nutrient dynamics, with implications for lake ecology and drinking water quality.

**Supplementary Information:**

The online version contains supplementary material available at 10.1007/s10533-024-01193-5.

## Introduction

**Fig. 1:**
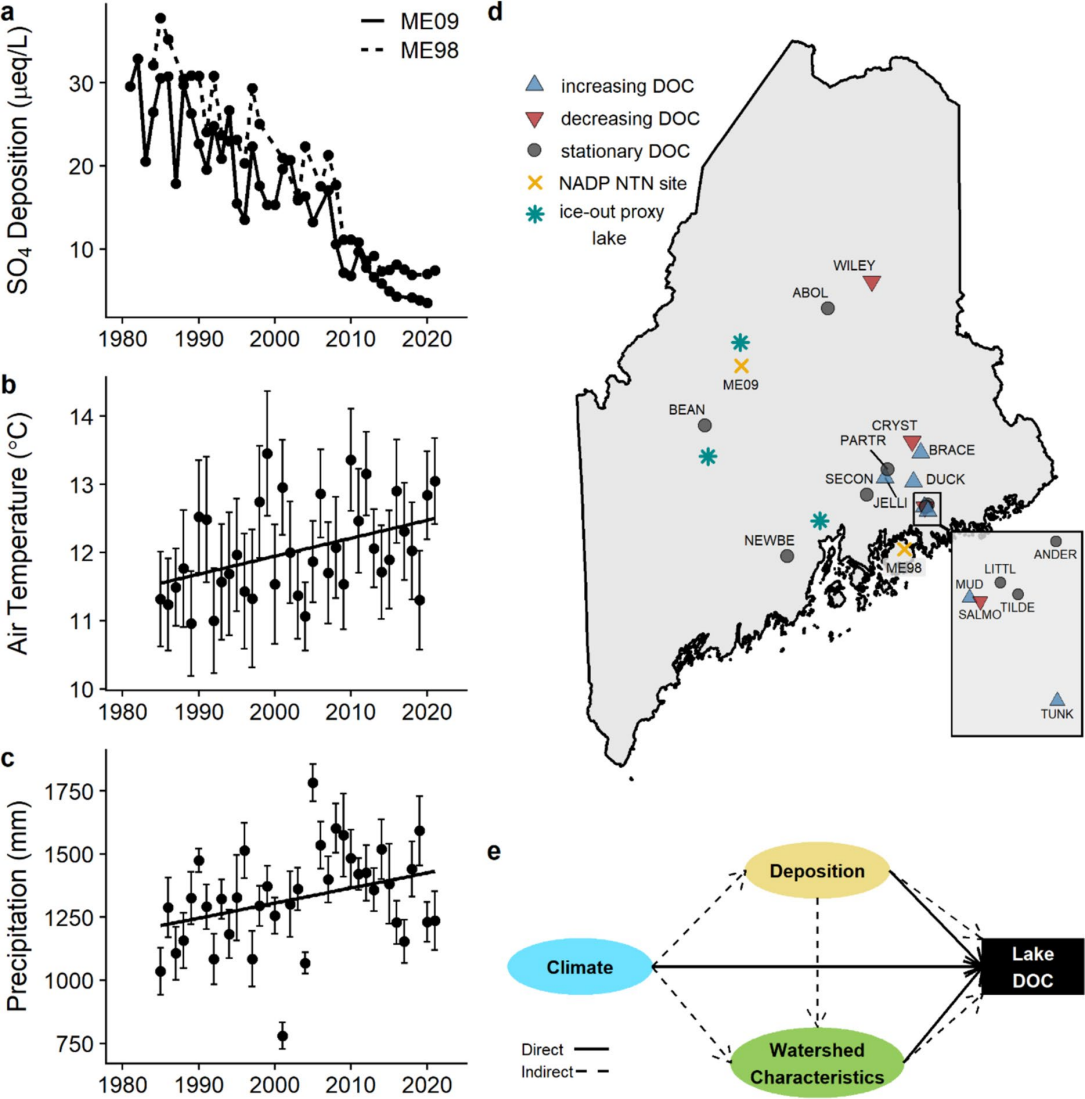
**a** Mean annual sulfate (SO_4_^2−^) wet deposition over time for the two Maine monitoring locations (ME09, solid line and ME98, dashed line) located near the study sites (**d**). **b** Mean annual air temperature in °C (R^2^ = 0.14, *p* < 0.05) and **c** total annual precipitation in mm (R^2^ = 0.09, *p* < 0.05) over time in the study region. Each point (**b, c**) represents the average across all lake sites and error bars denote standard deviation. **d** Study lakes colored by increasing (blue triangle), decreasing (red triangle) or stationary (grey circle) trend in dissolved organic carbon (DOC) over time (Supplementary Table 2). Deposition monitoring locations are labeled and denoted by a yellow X. Blue asterisks represent locations of ice-out proxy lakes. **e** Hypothesized direct (solid arrow) and indirect (dashed arrow) relationships between major drivers of lake DOC concentrations

The changing quantity and composition of surface water dissolved organic carbon (DOC) reflects complex interactions between watershed attributes and global change, such as atmospheric deposition and climate dynamics (McDowell 2023; Solomon et al. 2015; de Wit et al. 2021). Increasing surface water DOC concentrations, termed browning, have been observed in some freshwater ecosystems following reductions in atmospheric sulfur (S) deposition as a result of clean air policies (de Wit et al. 2007; Gavin et al. 2018; Monteith et al. 2007; Nelson et al. 2021; SanClements et al. 2012). Browning in lakes can impact provisioning of drinking water (Ledesma et al. 2012), increase greenhouse gas concentrations (Larsen et al. 2011) and alter lake thermal structure and light availability, which impacts habitat quality and lake productivity (Jane et al. 2024). Multiple facets of global change can influence lake chemistry, yet their relative contributions may vary through time due to changes in the relative importance of trends in atmospheric deposition (Gilliam et al. 2019; Murray et al. 2022) and climate (e.g., Meyer-Jacob et al. 2019; Räike et al. 2024; Fig. 1). Understanding how shifts in atmospheric inputs, increased air temperature and precipitation (Fig. 1) and shifting seasonality interact to drive lake DOC concentrations is crucial for predicting and managing the ecological consequences of browning in aquatic ecosystems.

Increases in surface water DOC in lake ecosystems that were historically impacted by acidic deposition have been largely attributed to the reduction of S concentrations in wet deposition (Monteith et al. 2007). Prior to regulation of S and reactive nitrogen (i.e., SO_2_, NO_x_) emissions, the acidification of soils resulted in higher sorption and decreased solubility of DOC because of an increase in soil ionic strength (Hruška et al. 2009; Monteith et al. 2007, 2023) which led to lower terrestrial inputs of DOC to receiving surface waters. As landscapes recover from acidic deposition, soils are becoming less acidic and lower in ionic strength. This landscape-scale shift has contributed to surface water browning by increasing DOC solubility and export (Monteith et al. 2007, 2023). The increase in DOC has significant biogeochemical implications, including alterations in carbon cycling, nutrient dynamics, and to light and thermal regimes of aquatic ecosystems. These changes can subsequently affect primary production, microbial activity, and the overall functioning of lake ecosystems (Jane et al. 2024).

The extent to which changes in soil ionic strength are attributed to recovery from acidification or climate warming remains unclear. For example, despite well-documented examples of increases in concentrations of DOC, some sites are showing declining surface water DOC concentrations and others exhibit stationary trends (Supplementary Fig. 1, Nelson et al. 2021; Rodríguez-Cardona et al. 2022; de Wit et al. 2021). This variability in temporal trends across broad spatial scales may be due to changes in climate drivers, such as temperature and precipitation, which can affect contemporary trends in lake DOC now that the forcing of sulfuric and nitric acid deposition has decreased (Gavin et al. 2018; de Wit et al. 2021). Disentangling the complex and variable effects of global change on lake C dynamics is crucial for understanding drivers of trends in DOC at intra-annual, inter-annual, and long-term scales and future changes in biogeochemical cycles.

As climate change progresses, many regions are becoming warmer, wetter, and experiencing shifting seasonality, with a loss of winter conditions commonly observed in areas with seasonal snow cover (Contosta et al. 2019). Warming winters in the northern hemisphere are causing increased frequency of freeze–thaw cycles in soils, resulting in increased leaching of organic matter (Wu et al. 2011). Additionally, changes in snowpack depth, the timing of snowmelt, and rain-on-snow events can alter runoff patterns and influence DOC flushing, with earlier snowmelt or increased rain-on-snow events potentially leading to earlier or more intense pulses of DOC into receiving waters (Contosta et al. 2017). Increases in air temperatures are expected to yield higher rates of terrestrial primary production and decomposition; yet simultaneous increases in soil respiration may lead to higher rates of removal from the same terrestrial carbon pool (Wu et al. 2011). Similarly, higher air temperatures can enhance soil base cation weathering by up to 9% per 1 °C rise in air temperature (Strang and Aherne 2015), which would counteract declines in soil ionic strength resulting from reduced S deposition.

Relatively remote lakes are ideal ecosystems for studying the consequences of changes in climate and deposition as their biogeochemistry is an integrated signal that reflects watershed-scale processes. The Clean Air Act (CAA) and associated amendments (1990) have reduced emissions of SO_2_ by 94% since 1990 (EPA 2021) resulting in decreases in surface water sulfate (SO_4_^2−^) concentrations and increases in pH and acid neutralizing capacity in many lakes (e.g., Strock et al. 2014). Despite the signs of recovery from the effects of acidic deposition, lakes have shown vulnerability to changes in climate, such as episodic acidification and browning during extreme dry and wet years, respectively (Strock et al. 2016). Changes in climate and seasonality, in combination with ongoing recovery from acidic deposition, may elicit spatially complex patterns in lake DOC concentrations.

In this study, we used 40 years of data on atmospheric deposition, climate, and surface water chemistry along with site-specific watershed characteristics from 16 well-studied lakes in Maine, USA to assess the interactive effects of global change and soil and watershed characteristics in driving changes in concentrations of DOC in northern temperate lakes. We hypothesized that the relative influence of atmospheric deposition would decline over time and that the relative importance of climate drivers (e.g., precipitation and temperature) would increase. After identifying persistent drivers of DOC over time, we further investigated the underlying biogeochemical mechanisms through which these drivers influence lake DOC dynamics. Specifically, we focused on two key aspects: (a) how watershed characteristics, such as soil properties and lake type, influence spatial patterns in lake DOC dynamics and (b) how shifting winter conditions, including changes in snow cover and precipitation phase, affect spring DOC concentrations. Navigating the complexities of interacting drivers of surface water DOC cycling underscores the need for comprehensive approaches to better understand and manage aquatic ecosystems in the context of changing environmental conditions.

## Materials and methods

Surface water chemistry data used in this study were collected as part of the New England Regional Long-Term Monitoring Program (RLTM) led by the Environmental Protection Agency (EPA). The primary objective at the onset of this monitoring program was to characterize the response of these aquatic systems to changes in atmospheric deposition and detect long-term trends in acid/base status following the Clean Air Act Amendments of 1990 (Kahl et al. 2004; SanClements et al. 2012; Stoddard et al. 2003).

### Site description and data collection

The RLTM project began in 1986 (for some lakes, sampling began as early as 1982 and as late as 1993) and includes 16 acid-sensitive lakes in Maine that are remote and/or have limited anthropogenic disturbance within their watershed boundaries (Stoddard et al. 1996). The lakes included in this study are relatively small (median lake area = 11.5 ha) and include 3 seepage and 13 drainage lakes (Table 1). Due to the remoteness of these lakes and the difficulty and danger of accessing them in the winter, the lakes were sampled once during the spring, summer, and fall seasons following standard EPA protocols for sample analysis (Hillman et al. 1986; Stoddard et al. 1996). Despite the limited intra-annual sampling frequency, these data are appropriate for assessments of long-term trends, as demonstrated by earlier studies (e.g., Kahl et al. 2004; SanClements et al. 2012; Stoddard et al. 2003).

**Table 1:** Lake and watershed characteristics for each site, collected as part of the Regionalized Long-Term Monitoring program led by the Environmental Protection Agency

Lake	Watershed area (ha)	Lake area (ha)	Site Elevation (m)	Max depth (m)	Lake type
Abol	1184	36	181	10	Drainage
Anderson	48	5	66	6	Drainage
Bean	141	12	381	9	Drainage
Bracey	91	8	117	9	Groundwater seepage
Crystal	32	10	113	9	Perched/seepage
Duck	47	2	82	3	Perched/seepage
Jellison Hill	223	18	123	17	Drainage
Little Long	217	24	75	25	Drainage
Mud	62	1	104	15	Drainage
Newbert	477	13	89	4	Drainage
Partridge	144	9	177	7	Drainage
Salmon	15	4	94	10	Drainage
Second	294	27	126	13	Drainage
Tilden	71	15	72	9	Drainage
Tunk (outlet)	4968	839	63	68	Drainage
Wiley	316	11	235	6	Drainage

Lake water samples were collected from the epilimnion at the deepest point of each lake at a depth of 0.5 m. Samples were collected by non-motorized boat in 500 mL high-density polyethylene (HDPE) bottles that were rinsed three times with lake water prior to collection. Aliquots from the unfiltered 500 mL bottle were decanted or filtered into smaller HDPE bottles within 48 h of collection and frozen or refrigerated until time of analysis. Samples were analyzed for dissolved organic carbon (DOC) and pH following standard EPA protocols and hold times (Peck 1992). Samples were analyzed at the University of New Hampshire, University of Maine, or Plymouth State University analytical laboratories following standard quality assurance protocols including external audit programs and uniform quality assurance evaluation across all project years (Hillman et al. 1986).

### Assessing changes in drivers of lake DOC

We used a two-step process to identify and quantify the major factors influencing lake DOC. The first step was to use structural equation modeling (SEM, e.g., Hair et al. 2021) to quantify the direct and indirect effects of climate, atmospheric deposition, and watershed characteristics on median lake DOC concentrations and to assess how the relative influence of drivers of DOC concentration changes over time. For the second step, with the objective of further understanding the mechanisms identified by the SEM results, we used multiple factor analysis (MFA) to explore spatial variability in watershed characteristics and mixed effect models to investigate the influence of winter conditions on spring DOC concentrations. More specific details about each approach are provided below.

We used structural equation modeling (SEM) to disentangle the influence of multiple drivers on an ecological response variable (e.g., Fazekas et al. 2023). Partial least squares structural equation modeling (PLS-SEM) can be used to define causal links and estimate the strength and direction of correlations between variables through an initial assessment of multivariate relationships between measured predictor variables (e.g., precipitation) and latent constructs (e.g., climate; Fan et al. 2016; Hair et al. 2021). Latent constructs represent an abstract concept that cannot be directly measured or defined by a single variable (e.g., climate, watershed characteristics). Partial least squares structural equation modeling is recommended for studies with small sample sizes and does not require specific assumptions about the data distribution or missing data making it ideal for analysis of datasets like ours (Fan et al. 2016).

Climate, deposition, and watershed characteristics represent the three latent constructs in the PLS-SEM, which were related to the predictor variables outlined in Supplementary Fig. 2. The significance of individual paths was determined using a *p* value threshold of 0.1. We specifically differentiated cases of marginal significance (*p* < 0.1) and cases where the *p* value was below 0.05. We used site-specific mean annual values for air temperature, precipitation, and atmospheric deposition, and median lake DOC concentration within each year for each site. Daily precipitation and air temperature were obtained from Daymet (Thornton et al. 2016) at a 1 km^2^ resolution allowing for site-specific averages of mean annual temperature (MAT) and precipitation (MAP) based on the latitude and longitude of each lake. Atmospheric wet deposition concentrations (NO_3_^−^, NH_4_^+^, and SO_4_^2−^ in µeq L^−1^) were obtained at an annual scale from the U.S. National Atmospheric Deposition Program/National Trends Network (NADP/NTN) from collection sites located closest to the study lakes (ME09 and ME98, Fig. 1d). Soil and watershed data were downloaded from the California Soil Resource Lab (Walkinshaw et al. 2022) at 800 m resolution for each site. Variables included drainage class, soil texture, and soil depth and are assumed to be fixed over the course of this study (Supplementary Table 1).

We analyzed the shifting role of atmospheric deposition, climate, and watershed characteristics on median DOC concentrations by splitting the data record (1985–2021) into four periods. The first period only includes a subset of lakes (*n* = 7) where sampling began in the 1980s. The first full period begins in 1993, the time at which all 16 lakes were sampled at the same frequency. This approach resulted in four models referred to as: Period 1 (1985–1992, *n* = 42), Period 2 (1993–2002, *n* = 134), Period 3 (2003–2012, *n* = 111), and Period 4 (2013–2021, *n* = 121). We used the SmartPLS 4 software to build the PLS-SEM models (Ringle et al. 2024).

### Mechanistic interpretation of dominant drivers of lake DOC patterns

After identifying the dominant drivers through PLS-SEM, we use additional statistical analyses to determine how these drivers influence DOC concentrations across different spatial and temporal scales. Specifically, we used multiple factor analysis to explore spatial variability in watershed characteristics (section "Watershed characteristics") and mixed-effect modeling to investigate the impact of changing winter on spring DOC concentrations (section "Winter climate").

#### Watershed characteristics

We used multiple factor analysis (MFA, using *FactoMineR* in R, Lê et al. 2008) coupled with simple linear regression to infer the mechanism by which watershed characteristics influence lake DOC dynamics across sites with varying directionality of temporal trends (Supplementary Fig. 1). Multiple factor analysis is appropriate for these data because it can handle collinearity among predictor variables and allows for the integration of both quantitative and qualitative data. For the MFA, we included the following quantitative predictor variables for each site: latitude, site elevation, watershed area to lake area ratio (WA:LA), soil texture, soil depth, and maximum lake depth. Drainage class (e.g., well drained, poorly drained) and lake type (drainage vs. seepage) were included as qualitative predictor variables (Supplementary Table 1; Table 1). We used the first two axes (Dim1, Dim2) in linear regression to determine how much within-site variability in DOC, as measured by the coefficient of variation (CV), could be explained by these factors.

#### Winter climate

We specifically focused on exploring the role of winter conditions on DOC dynamics because winter has undergone the most substantial changes of all seasons in our study area (Fernandez et al. 2020) and remains understudied, especially with respect to potential cascading effects on subsequent seasons (Hampton et al. 2017). We subset climate data from December through March and calculated mean winter air temperature, cumulative winter precipitation, the number of snow-covered days, and the proportion of winter precipitation falling as snow (expressed as snow %, see Supplementary Materials). Winter was defined as December through March based on which months had been previously designated as spring (April-June) and fall sampling (October–November). The number of snow-covered days was calculated as the sum of days where snow water equivalent was greater than 0 km m^−2^ (Contosta et al. 2019). Whether winter precipitation fell as rain or snow was determined using regional air temperature thresholds obtained from Jennings et al. (2018) for individual lakes: 0.56 °C for Newbert, 0.67 °C for Abol and Wiley, and 0.90 °C for the remaining sites. These region-specific air temperature thresholds are the 50% rain-snow threshold, representing the temperature at which precipitation falls as rain and snow equally, accounting for local geographic and climatic variation. Precipitation below these temperatures is assumed to fall as snow (Jennings et al. 2018).

Ice-out data through 2008 were obtained from the United States Geological Survey (USGS) and from the Maine Department of Agriculture, Conservation and Forestry for 2008 onward. Lake ice-out dates refer to the date when a given water body is free of winter ice cover and is navigable from one end of the water body to the other. Data from both sources were compiled from town officials, lake associations, fisheries agencies, and Maine residents. We chose three lakes with the most complete, long-term ice-out records as proxies for the lakes in this study, as ice-out dates have not been consistently reported at our sites. We used data from Moosehead Lake to represent the northern sites (Abol and Wiley), Embden Pond to represent the western-most site (Bean), and Swan Lake as a proxy for the remaining coastal sites.

We assessed the influence of winter conditions on spring DOC concentrations using mixed-effect models (using *lme4* in R, Bates et al. 2015). Winter climate indicators (described below and in Supplementary Table 2) were included as fixed effects, and we tested various random structures, including lake, year, and nested lake-year effects, and selected the best-fitting structure based on Akaike information criterion (AIC) values (Akaike 2011). Fixed effect predictor variables were excluded in a stepwise backward direction, using stepwise multiple regression, when alternative models resulted in lower AIC values. We reported the amount of variance explained by fixed effects alone (marginal R^2^, R^2^_m_) and by the combined fixed and random effects (conditional R^2^, R^2^_c_) using the MuMIn package in R (Bartoń 2023; Nakagawa and Schielzeth 2013). The level of significance (α) was set to 0.05.

## Results

### Drivers of lake DOC concentration over time

**Fig. 2:**
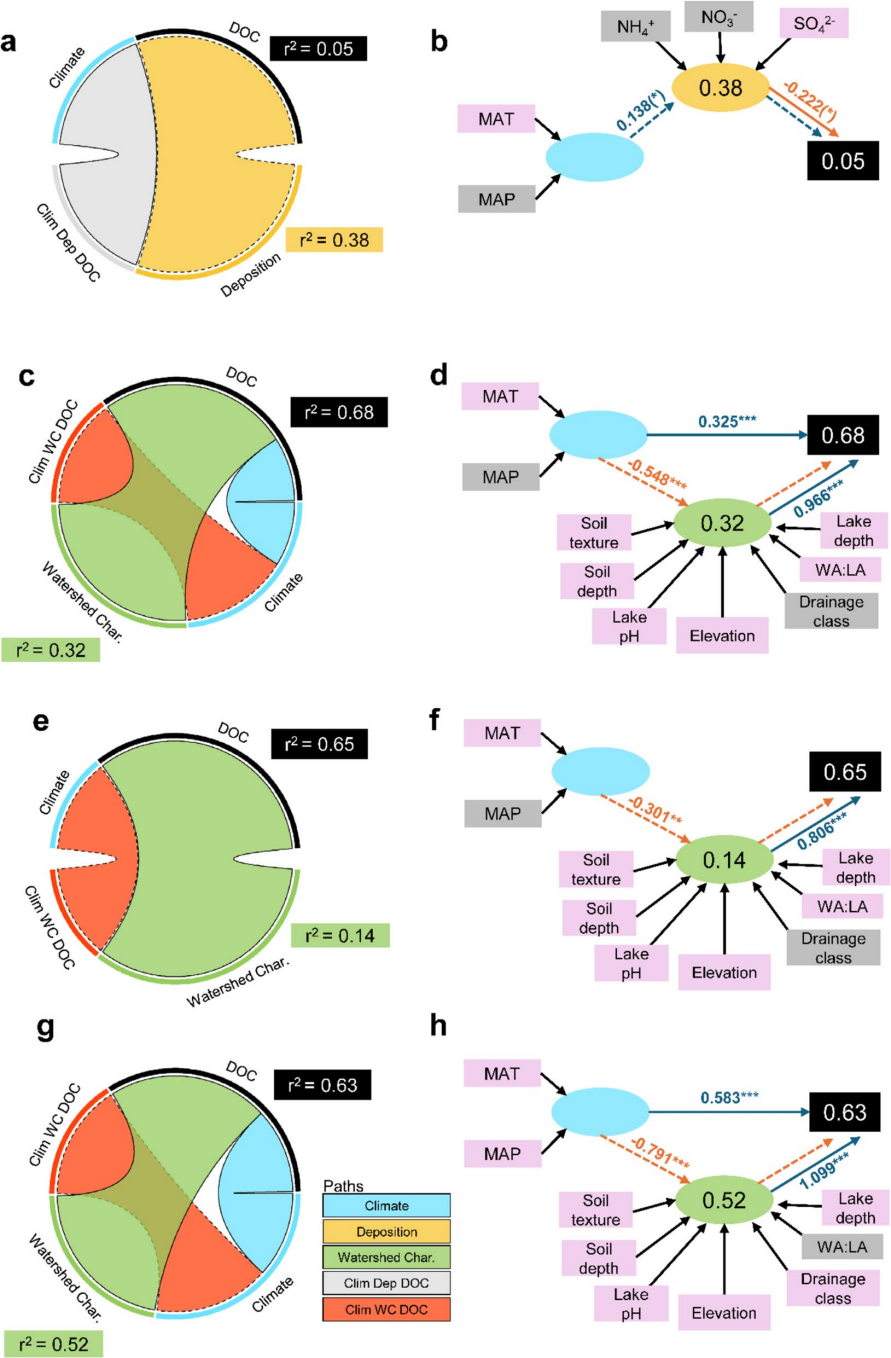
Chord diagrams (left) representing the results of PLS-SEM path relationships driving lake DOC across four periods: **a, b** Period 1 (1985–1992), **c, d** Period 2 (1993–2002), **e, f** Period 3 (2003–2012), and **g, h** Period 4 (2013–2021). Chord diagram paths are directional from either Climate, Deposition, or Watershed Characteristics directly to DOC (blue, yellow, and green bands) or via an indirect path (red and grey bands). Solid arcs represent positive path coefficients and dashed arcs indicate negative path coefficients. In (**b**), (**d**), (**f**), and (**h**) blue arrows indicate positive relationships and orange indicates negative relationships. Dashed arrows indicate indirect paths and solid lines represent direct paths. Values next to the arrows are path coefficients (standardized partial regression coefficients) with associated statistical significance (***P < 0.001; **P < 0.01; *P < 0.05; (*) *P* < 0.1). Values within the latent constructs represent the percentage of variance explained by the model. Indicators colored in light purple load significantly on the respective latent construct

Watershed characteristics were the predominant factor driving DOC over time except Period 1. Consistent with our hypothesis, the relative influence of atmospheric deposition and climate to drive changes in lake DOC concentrations shifted across the 40-year sampling record (Fig. 2). Sulfate deposition had a small, marginally significant influence on lake DOC concentrations in the 1980s, coinciding with peak levels of SO_4_^2−^ deposition (Figs. 1a and 2a–b). Climate consistently influenced DOC concentrations over time, both directly and indirectly, with the strongest effect observed in Period 4 (2013–2021; Fig. 2c–h).

In Period 1 (1985–1992, Fig. 2a–b), the PLS-SEM model explained only 5% of the total variance in median DOC. Deposition had a direct, negative effect (path coefficient [β] =  − 0.222, *p* = 0.085) and was only indicated by SO_4_^2−^ deposition. The indirect pathway between climate and DOC through deposition positively predicted DOC (β = 0.138, *p* = 0.102) and was indicated by mean annual air temperature. It is important to note that the lower number of lakes in this period (7 instead of 16) likely contributes to the reduced explanatory power and significance of the model, and these results should therefore be interpreted with these limitations in mind.

The amount of variance explained in the following period (Period 2: 1993–2002, Fig. 2c–d) increased to 68%. Watershed characteristics had a direct, positive effect on DOC (β = 0.966, *p* < 0.0001) as did climate (β = 0.325, *p* < 0.0001). The indirect pathway between climate and DOC through watershed characteristics negatively predicted DOC concentration (β = − 0.548, *p* < 0.0001). Lake characteristics were strongly indicated by soil texture (0.744, p < 0.0001), site elevation (0.595, *p* < 0.0001), soil depth (0.503, *p* < 0.0001), lake pH (− 0.298, *p* = 0.0002), WA:LA (− 0.199, *p* = 0.003), and lake depth (− 0.114, *p* = 0.005). Climate was significantly indicated by mean annual air temperature (1.00, *p* < 0.0001).

Pathways were similar in Period 3 (2003–2012, Fig. 2e–f) except for the lack of a direct effect of climate. Watershed characteristics had a direct, positive effect on DOC (β = 0.806, *p* < 0.0001) and the indirect pathway between climate and DOC through watershed characteristics negatively predicted DOC (β = − 0.301, *p* = 0.0012). Together these pathways explained 65% of the total variance in median DOC. Lake characteristics were strongly indicated by soil texture (0.831, *p* < 0.0001), lake pH (− 0.562, *p* < 0.0001), site elevation (0.490, *p* < 0.0001), soil depth (0.467, *p* < 0.0001), WA:LA (− 0.287, *p* < 0.001), and lake depth (− 0.089, *p* = 0.022). Climate was significantly indicated by mean annual air temperature (1.00, *p* < 0.0001).

Lastly, the PLS-SEM model for Period 4 (2013–2021, Fig. 2g–h) explained 63% of the total variation in median DOC. Both watershed characteristics and climate had a direct, positive effect on DOC (watershed characteristics β = 1.099, climate β = 0.583; both *p* < 0.0001). The indirect pathway between climate and DOC through watershed characteristics negatively predicted DOC (β = − 0.791, *p* < 0.0001). Lake characteristics were strongly indicated by site elevation (0.584, *p* < 0.0001), soil texture (0.566, *p* < 0.0001), soil depth (0.505, *p* < 0.0001), lake pH (− 0.254, *p* < 0.0001), lake depth (− 0.114, *p* = 0.002) and drainage class (0.074, *p* = 0.052). In contrast to the previous periods, climate was significantly indicated by both mean annual air temperature (0.923, *p* < 0.0001) and mean annual precipitation (0.648, *p* < 0.0001).

### Variability in watershed characteristics

**Fig. 3:**
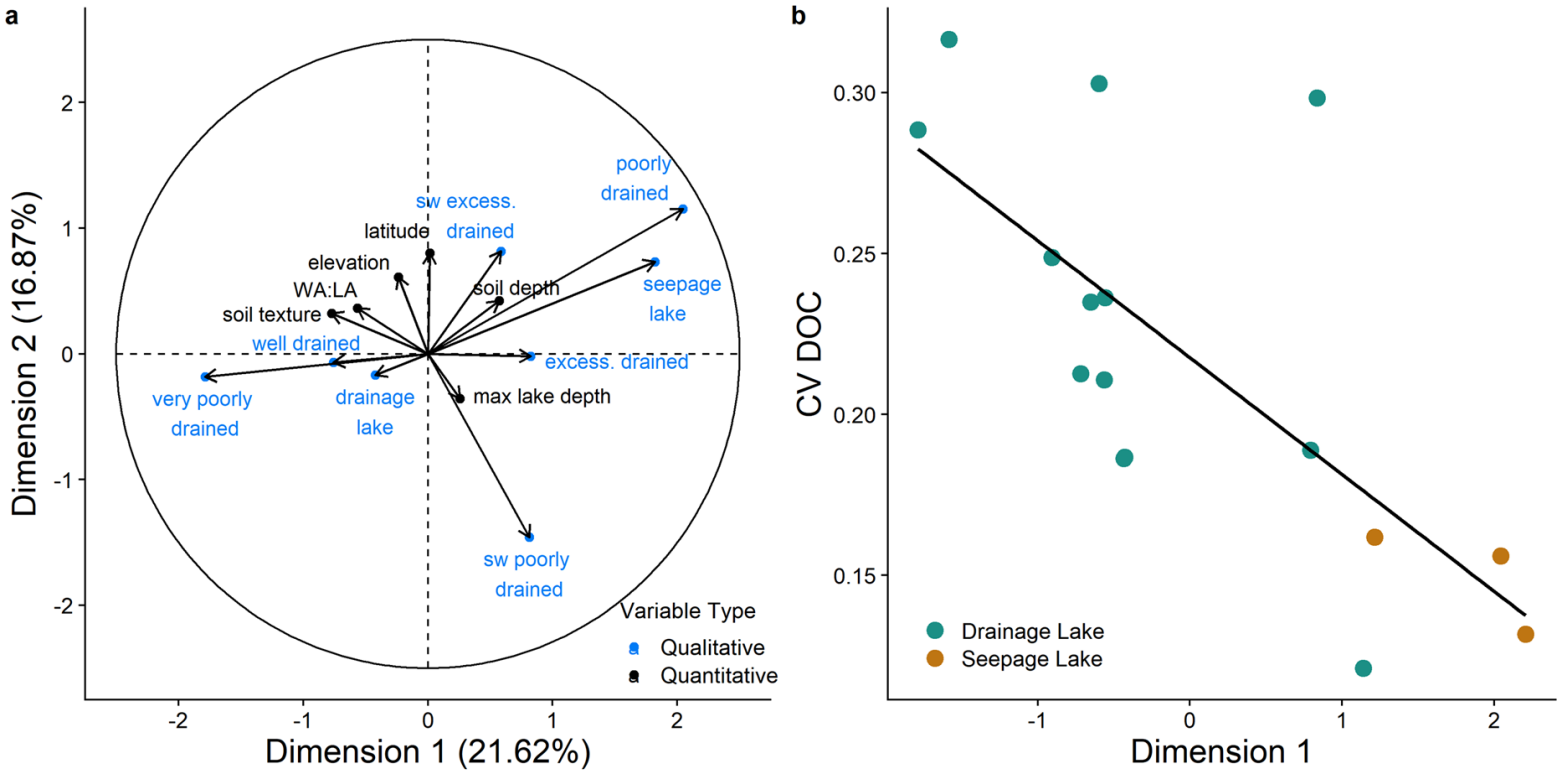
**a** Results of Multiple Factor Analysis (MFA) of watershed characteristic predictor variables. Colors denote qualitative (blue) and quantitative (black) variables. **b** Linear regression between the first axis (Dimension 1) of the MFA describing watershed characteristics and the coefficient of variation in dissolved organic carbon (CV DOC, R^2^ = 0.48, *p* = 0.001825). Each point represents the CV in DOC for each lake. Colors denote lake type. No additional variation in CV DOC was accounted for by Dimension 2 (Supplementary Fig. 3)

The first two dimensions (Dim1 and Dim2) of the MFA explained 38.5% of the variance in watershed characteristics (Fig. 3a). Among the quantitative variables, soil depth contributed significantly and positively to Dim1 (0.57, *p* < 0.05) while soil texture (− 0.77, *p* < 0.05) and watershed to lake area ratio (WA:LA, − 0.56, *p* < 0.001) contributed significantly and negatively to Dim1. For Dim2, latitude (0.80, *p* < 0.001) and site elevation (0.61, *p* < 0.05) were significant, positive contributors. No predictor variable contributed negatively to Dim2.

Both qualitative variables, lake type (*p* < 0.001) and drainage class (*p* < 0.05), contributed significantly to Dim1. Within the lake type category, seepage lakes contributed significantly and positively to Dim1 (1.12, *p* < 0.001) while drainage lakes contributed significantly and negatively (− 1.12, *p* < 0.001). Despite the significant contribution from the drainage class category, only one of the six drainage categories (well-drained) was a significant contributor to Dim1 (− 1.04, *p* < 0.05). The first MFA dimension (Dim1) correlated negatively with the coefficient of variation of DOC concentrations (R^2^ = 0.48, *p* < 0.01; Fig. 3b). No additional variation in CV DOC was explained by Dim2 (Supplementary Fig. 3).

### Impact of winter on spring DOC concentrations

Results of the mixed-effect model show that the proportion of winter precipitation falling as snow, in combination with site:year as a random effect, explain 99% of the variation in spring DOC concentrations (Table 2). This random structure allows us to account for both inter-annual variability and lake-specific effects on DOC concentrations, providing a more robust understanding of the relationship between winter conditions and spring DOC levels. Other winter climate indicators such as mean winter air temperature, number of snow-covered days, and cumulative winter precipitation did not significantly improve the model and were removed. We found that rainier winters (a decreased percentage of winter precipitation falling as snow) led to increased concentrations of lake DOC in the following spring as compared to snowier winters (*β* = − 0.14, Table 2). The inclusion of site:year as a random effect in our model increased the explanatory power (from R^2^_m_ = 0.005 to R^2^_c_ = 0.99, Table 2), suggesting that both annual variability and unmeasured site-specific characteristics are crucial in explaining DOC dynamics.

**Table 2:** Results from linear mixed-effects models with fixed effects and the random effect of site:year (not shown) for spring DOC concentrations in lakes

Response	Fixed Effects	*β*	R^2^	
			R^2^_m_	R^2^_c_
Spring DOC	Snow (%)	− 0.14	0.005	0.99

*β* values indicate the strength and directionality of each predictor in the model. Variation explained by fixed effects alone is shown by the marginal coefficient of determination (*R*^2^_m_), and the conditional coefficient of determination (*R*^2^_c_) accounts for the variation explained by both fixed and random effects

## Discussion

Using a 40-year record from remote lakes in a northern temperate biome, we document how DOC concentrations in lakes historically impacted by acidic deposition are now influenced primarily by climate factors. This shift reflects a transition from a primary forcing by atmospheric deposition to the increasing role of climate drivers, like temperature, precipitation, and precipitation phase, in the last ten years. Watershed characteristics, such as soil texture and depth, also play a significant role in shaping DOC concentrations by regulating within-site DOC variability and modulating the impact of climate.

### Shifts in drivers of lake DOC concentrations over time

Our PLS-SEM results reveal a temporal shift in the dominant global change driver of lake DOC concentrations, moving from an early influence of acidic deposition to a period increasingly controlled by climate factors. Throughout our study period, watershed characteristics persisted as key determinants of DOC concentrations over time, both directly and indirectly. It is well-established that factors like terrestrial vegetation, soil properties, land cover, and hydrology dictate ranges of DOC concentrations in lakes (Sobek et al. 2007). Consistent with this, our analysis shows that the effects of climate on lake DOC concentrations are frequently moderated or accentuated by watershed characteristics (Fig. 2 and Table 2).

Soil texture, soil depth, site elevation, and lake pH were consistent, significant indicators of watershed characteristics and subsequently DOC concentrations (Fig. 2). The positive influences from soil texture, depth, and elevation on the watershed characteristic construct, and the subsequent positive relationship between this construct and DOC concentrations, suggests that watersheds with finer-textured soils, deeper soil layers, and higher elevations contribute more organic carbon (OC) to lakes. This pattern is consistent with findings that watershed soil carbon density and OC stocks are positively related to lake DOC, with the magnitude of export from soils to lakes dependent on other soil parameters such as soil depth, productivity rates, and water flow paths specific to each watershed (Sobek et al. 2007). Consistent trends in DOC over time have been observed across high elevation mountain ponds (Nelson et al. 2021; Gavin et al. 2018), suggesting that responses to drivers of DOC may differ among lakes in different climate regions (Fazekas et al. 2023) due to variability in topography and soil characteristics. The significant influence of lake pH likely reflects the role of pH in regulating the watershed-scale solubility of terrestrial OC and the subsequent magnitude of OC that can be mobilized from soil to surface water, a dynamic that has shifted over time with the regional increase in pH, likely driven by declining SO_4_^2−^ deposition (Monteith et al. 2007, 2023; Fig. 1a).

Climate variability and the increased frequency and intensity of weather events may result in significant impacts to northern lake DOC concentrations, with implications for water quality and ecosystem function. Watersheds may, however, buffer the impact of climatic influences. Our analyses highlight the importance of interactions between climate and soil and watershed properties (Fazekas et al. 2023), with the indirect influence of climate through watershed characteristics being consistently stronger than the direct influence of climate on lake DOC concentrations in our SEMs (Fig. 2). Climate is an important regulator of terrestrial OM stocks through enhanced primary productivity at higher temperatures (Cao and Woodward 1998) or by facilitating greater export of DOC to receiving waters following large precipitation events, or retention of C on the terrestrial landscape during dry periods (Sobek et al. 2007; Strock et al. 2016). The consistent contribution of mean air temperature to climate from Period 1 through 4, coupled with precipitation becoming a significant contributor in the most recent period, underscores the growing role of climate variables in shaping lake chemistry, and highlights how changes in climate are increasingly affecting lake DOC. Climate has likely always played a crucial role in shaping lake DOC. However, the diminishing influence of other drivers, such as acidic deposition, in combination with recent alterations in precipitation frequency and form, as well as temperature fluctuations, may contribute to the reemergence of climate as a dominant forcing factor (Meyer-Jacob et al. 2019). The net effect of climate on DOC will vary based on localized hydrology, projected changes in vegetation, and soil properties (Solomon et al. 2015), underscoring the importance of considering how site-specific landscape and physical lake characteristics influence patterns in DOC both directly and indirectly.

Shifting patterns of biogeochemical cycles as a function of warming winters is a phenomenon that has been quantified across the continental United States (Seybold et al. 2022). The Northeastern United States is becoming warmer and wetter, with annual air temperatures in Maine increasing by 1.8 °C since 1895 and projected to warm by an additional 1.1–2.2 °C by 2050 (Fernandez et al. 2020; MCC STS 2020). The increasing influence of climate over time (Fig. 2) may be explained by the intensification of climatic cycles. Statewide annual precipitation in Maine has increased by 152 mm, marked by more frequent heavy storms and increased flood volumes (MCC STS 2020). Winter has warmed the most of all seasons, with a higher proportion of winter precipitation falling as rain in New England, which is expected to continue as the snow-rain transition zone shifts northward (Dupigny-Giroux et al. 2018). The loss of winter is evidenced by a decline in snowpack depth, fewer snow-covered days (Contosta et al. 2019), and earlier ice-out on lakes (Fernandez et al. 2020), accompanied by reduced ice thickness and duration in recent decades (MCC STS 2020). Strong links have been made between changes in winter conditions and nutrient dynamics in lakes (e.g., Blank et al. 2009; Hampton et al. 2017), with changes to the timing and magnitude of snowmelt, species phenology, and annual surface water budgets potentially contributing to observed changes in nutrient availability. Previous work shows that inter-seasonal connections are common for biogeochemical and ecological variables (Hampton et al. 2017) and that changing winter conditions can have cascading effects on lake chemistry and ecosystem function in subsequent seasons.

### Rainier winters lead to higher spring DOC concentrations

The diminution of winter has important yet understudied consequences for energy and nutrient balances in both terrestrial and aquatic ecosystems (Contosta et al. 2017; Hampton et al. 2017; Seybold et al. 2022). Our results suggest that as winters continue to warm and the dominant precipitation phase shifts from snow to rain, lakes are likely to receive greater inputs of terrestrial DOC in the spring (Table 2). Previous work has shown that declines in lake ice cover have altered winter N dynamics, with reduced ice duration potentially leading to lower nitrate accumulation under ice (Powers et al. 2017), suggesting that shorter ice cover periods could diminish an important source of N for low-nutrient systems like the lakes in our study. Our findings that warmer, rainier winters are associated with increased spring DOC concentrations suggests a potential shift in the balance of C and N inputs to these systems.

The inclusion of the random structure of site and year in the LMM was crucial in explaining the observed variation in DOC concentrations, capturing the influence of both localized site-level differences and temporal changes over time. The role of localized landscape and lake characteristics, such as elevation and soil attributes, in regulating the impact of winter climate on spring DOC concentrations is also supported by the SEM results, which highlight the mediating role of watershed characteristics on the effect of climate on DOC concentrations.

The interactions between changing C and N availability due to shifts in winter conditions will play a key role in modifying seasonal and annual nutrient and energy balances in lakes, with implications for biogeochemical cycling and overall ecological health. For example, future spring DOC increases may contribute to changes in lake thermal structure potentially affecting the resiliency of cold-water refugia (Gavin et al. 2023), and concurrent changes in other chemical properties may influence zooplankton communities and food web dynamics (Dykema et al. 2023). Considering the impact of warming winters and the increased occurrence of weather whiplash (Loecke et al. 2017) on lentic DOC dynamics is of particular importance in this region where warming winters and increased climate variability are now the norm and exert a strong influence on patterns in lake chemistry.

### Importance of watershed characteristics in regulating DOC variability

Watersheds characterized by fine-textured, shallow soils and higher watershed to lake area ratios (WA:LA) are associated with greater variability in lake DOC concentrations. This suggests that watersheds with deeper, coarser-textured soils may act as a buffer against the mobilization of terrestrial OC resulting from precipitation events, as coarser soils facilitate more rapid water infiltration, reducing surface runoff and limiting the transport of OC to aquatic systems (McDowell 2023; Solomon et al. 2015). This buffering capacity likely stabilizes lake DOC concentrations by promoting more consistent retention and transport of soil OM. Drainage lakes and those with higher watershed area to lake area ratios displayed greater variability in DOC concentrations compared to seepage lakes, likely reflecting increased terrestrial-aquatic connectivity and more variable surface flows in drainage lakes (Erlandsson et al. 2008). Additionally, lakes draining larger watersheds (larger WA:LA) receive additional sources and volumes of runoff, providing greater opportunities for leaching of OM and thus contributing to more variability in DOC concentrations.

The importance of site-level characteristics in regulating aquatic DOC concentrations highlighted by our results is often missing in larger spatial assessments of factors controlling trends in DOC (Fazekas et al. 2023). Understanding how watershed characteristics drive lake DOC dynamics provides valuable insights into the complex interactions shaping DOC concentrations across heterogeneous landscapes. Our results show that incorporation of site-specific watershed characteristics will be important for better predicting future trends and variability in lake DOC concentrations (Supplementary Fig. 1). As precipitation events intensify, the export of DOC to lakes may vary based on soil properties and topography, creating challenges for predicting overall changes in surface water DOC concentrations without consideration of site-level nuances. Understanding the interplay of watershed characteristics is essential for effectively managing and predicting lake DOC concentrations under changing climate patterns and deposition levels.

## Conclusions

The increasing and strengthening influence of climate on lake DOC over time underscores the importance of considering both short- and long-term climate impacts on lake chemistry, particularly in the context of warming winters and increased climate variability. Our results also emphasize the critical role of localized watershed features, such as soil texture and depth, elevation, and lake type, in modulating DOC impacts from climate. These characteristics influence the transport and retention of organic carbon in ways that impact variability in DOC concentration across lakes (Fig. 3, Supplementary Fig. 1). Given the variability in directionality of temporal DOC trends across freshwater ecosystems (Nelson et al. 2021; Rodríguez-Cardona et al. 2022; Fig. 1, Supplementary Fig. 1), it is important for future studies to integrate site-specific watershed characteristics and climate dynamics to better understand and predict trends in DOC concentrations across diverse lake ecosystems which remain vulnerable to climate change. A nuanced understanding of DOC and climate dynamics at the individual-lake scale is important for water resource management, as browning can impact drinking water supply (Ledesma et al. 2012), alter food web dynamics (Dykema et al. 2023), and eliminate cold-water fish habitat (Jane et al. 2024), with simultaneous impacts to overall lake productivity and biogeochemical cycling.

## Supplementary Information

Below is the link to the electronic supplementary material.

Supplementary file1 (DOCX 677 KB)

## Data Availability

Atmospheric deposition data are available from the National Atmospheric Deposition Program National Trends Network (https://nadp.slh.wisc.edu/networks/national-trends-network/). Climate data (temperature, precipitation, SWE) can be obtained from Daymet (https://daymet.ornl.gov/). Ice-out data can be obtained from the United States Geological Survey (*Historical Ice-Out Dates for 29 Lakes in New England, 1807–2008: USGS Open-File Report 2010–1214*. http://pubs.usgs.gov/of/2010/1214/) and the Maine Department of Agriculture, Conservation and Forestry (https://www.maine.gov/dacf/parks/water_activities/boating/ice_out_dates.shtml). Gridded soil attributes can be downloaded from the California Soil Resource Lab (https://casoilresource.lawr.ucdavis.edu/soil-properties/). Other watershed attributes collected as part of the EPA RLTM program can be found in Table 1. Water chemistry from the RLTM program can be downloaded from the EPA website (https://www.epa.gov/power-sector/monitoring-surface-water-chemistry).
