# Supplementary material for: Climate displaces deposition as dominant driver of dissolved organic carbon concentrations in historically acidified lakes
Source: Biogeochemistry. 2024 Dec 21;168(1):5. doi: 10.1007/s10533-024-01193-5 (PMC11662036; doi:10.1007/s10533-024-01193-5)
Supplement: Supplementary file 1 — Supplementary file1 (DOCX 677 KB) [file 10533_2024_1193_MOESM1_ESM.docx]

*Supplementary Materials for the manuscript:*

**Climate displaces deposition as dominant driver of dissolved organic carbon concentrations in historically acidified lakes**

Allison M. Herreid^1,2^, Hannah M. Fazekas^1,3^, Sarah J. Nelson^4^, Adam S. Wymore^1^, Desneiges Murray^1^, Ruth K. Varner^5^, William H. McDowell^1^

^1^Department of Natural Resources and the Environment
56 College Road
University of New Hampshire, Durham, NH, USA

^2^U.S. Department of Agriculture – Agricultural Research Service
Soil and Water Management Research
1991 Upper Buford Circle
University of Minnesota, St. Paul, MN, USA
(*current affiliation)*

^3^Biology Department
Saginaw Valley State University
University Center, MI, USA
(*current affiliation)*

^4^Appalachian Mountain Club, Research Department
361 Route 16
Gorham, NH 03581

^5^Department of Earth Sciences and the
Institute for the Study of Earth, Oceans, and Space
8 College Road
University of New Hampshire, Durham, NH, USA

**Introduction**

The supplementary materials provide additional methodological details and results that support and extend the findings discussed in the main manuscript. Specifically, it contains the approach used to assess trends in dissolved organic carbon (DOC) concentrations over time at individual lake sites using non-parametric statistical tests. Supplementary tables offer key information on the fixed effects included in the linear mixed-effects models and their sources, along with detailed site-level soil characteristics for each lake in the study. Supplementary figures illustrate the initial partial least squares structural equation model and relationship between the coefficient of variability in DOC and the second axis of the multiple factor analysis.

**Supplementary Methods**

To evaluate the existence, direction, and magnitude of a significant trend in dissolved organic carbon (DOC) over time at each site (Figure 1; Supplementary Figure 2), we used the non-parametric Mann-Kendall and Sen slope tests using the *trend* package in R (Pohlert, 2023). Significant trends were identified when both Mann-Kendall and Sen slope tests had *p* < 0.05. The Sen slope test generated slope values for significant trends, indicating the median change in DOC over time.

Pohlert, T. (2023). *trend: Non-Parametric Trend Tests and Change-Point Detection* (1.1.6) [Computer software]. https://cran.r-project.org/web/packages/trend/index.html

**Supplementary Table 1.** Site-level soil characteristics (800 m resolution) obtained from the California Soil Resource Lab (Walkinshaw, 2022). Soil texture values are defined as: 2: loamy sand, 3: sandy loam, 4: loam, 5: silt loam. Soil depth is reported in centimeters.

| **Lake** | **Drainage Class** | **Soil Texture** | **Soil Depth (cm)** |
| --- | --- | --- | --- |
| Abol | well drained | 3.7 | 163.4 |
| Anderson | well drained | 4 | 69.7 |
| Bean | well drained | 3.3 | 144.2 |
| Bracey | poorly drained | 3.3 | 169.5 |
| Crystal | excessively drained | 2 | 165 |
| Duck | somewhat excessively drained | 3.3 | 165 |
| Jellison Hill | well drained | 3.3 | 107.1 |
| Little Long | somewhat poorly drained | 3 | 156.3 |
| Mud | well drained | 4 | 87.3 |
| Newbert | very poorly drained | 5 | 141.1 |
| Partridge | excessively drained | 4 | 54.0 |
| Salmon | well drained | 4 | 87.3 |
| Second | well drained | 3.3 | 107.9 |
| Tilden | somewhat poorly drained | 3 | 156.3 |
| Tunk (outlet) | somewhat excessively drained | 3 | 162.8 |
| Wiley | somewhat excessively drained | 5 | 178.4 |

**Supplementary Table 2.** Initial linear mixed-effects fixed effects with definitions and data sources and references.

| **Response** | **Fixed Effect** | **Definition** | **References** |
| --- | --- | --- | --- |
| Spring DOC | Snow (%) | Proportion of winter precipitation falling as snow; determined using regional air temperature thresholds | Daymet; Jennings et al., 2018 |
|  | Mean winter air temperature (°C) | Mean air temperature from December through March | Daymet |
|  | Total winter precipitation (mm) | Total precipitation from December through March | Daymet |
|  | Number of snow-covered days | sum of days where SWE > 0 | Daymet; Contosta et al. 2019 |
|  | Ice-out date | The date when a lake is free of winter ice cover and navigable from end to end | USGS; Maine Department of Agriculture, Conservation and Forestry |


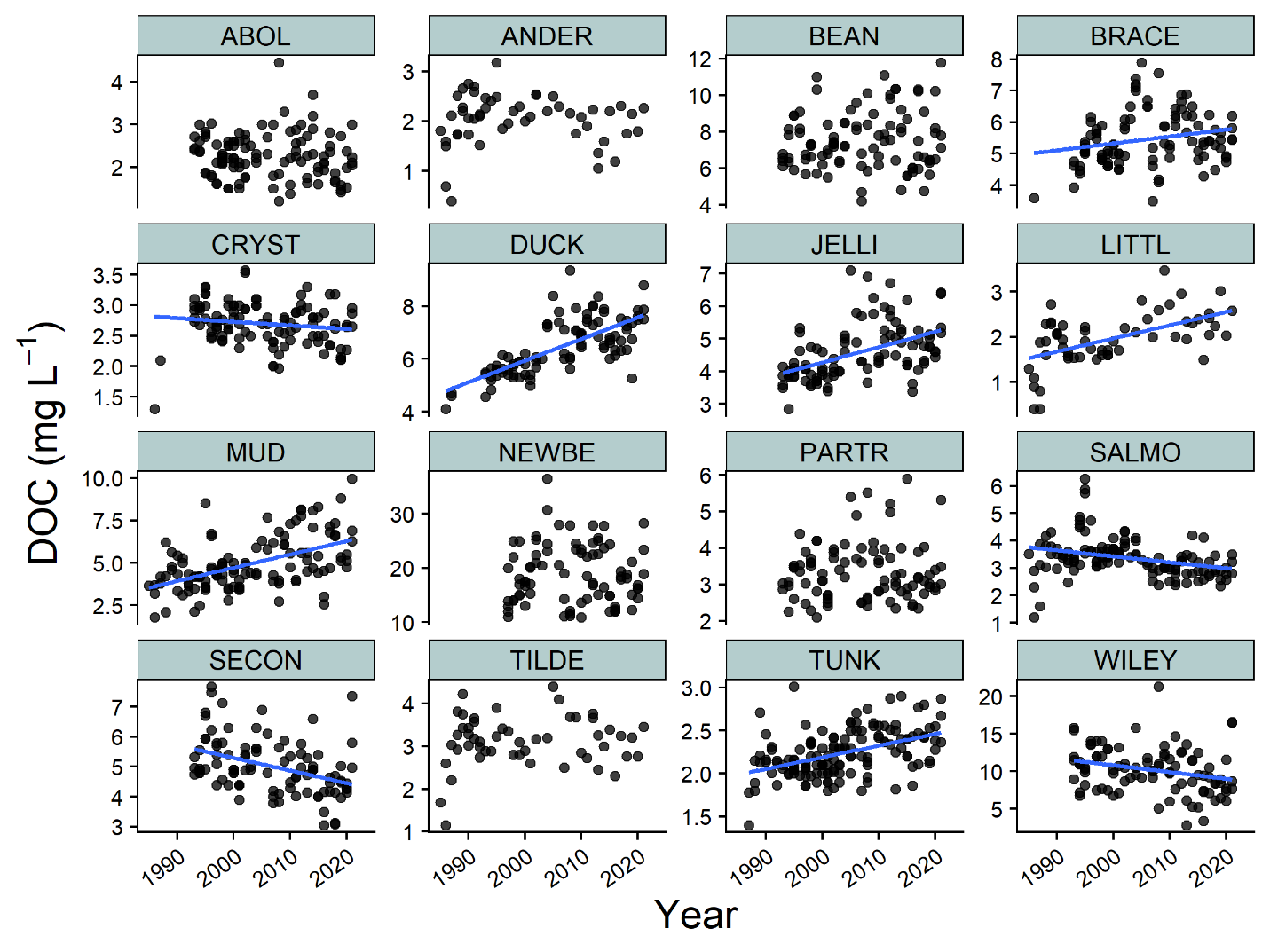
 **Supplementary Figure 1.** Trends in dissolved organic carbon (DOC in mg L^-1^) over the period of record for each lake. Blue lines indicate significant sen slopes.


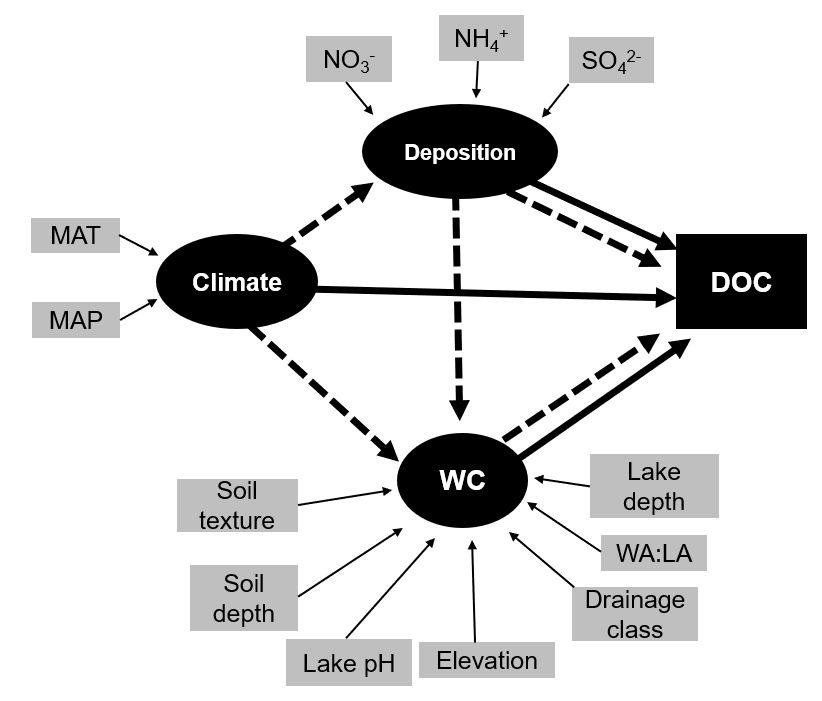


**Supplementary Figure 2.** Initial partial least squares structural equation model structure with hypothesized direct (solid arrow) and indirect (dashed arrow) causal relationships between major components (circles) driving lake dissolved organic carbon (DOC) concentrations. Circles represent latent constructs (Climate, Watershed Characteristics [WC], Deposition) which are influenced by manifest (measured predictor) variables which are represented by the grey boxes. Abbreviations are as follows: MAT = mean annual air temperature, MAP = mean annual precipitation, WA:LA = watershed area to lake area ratio.

**
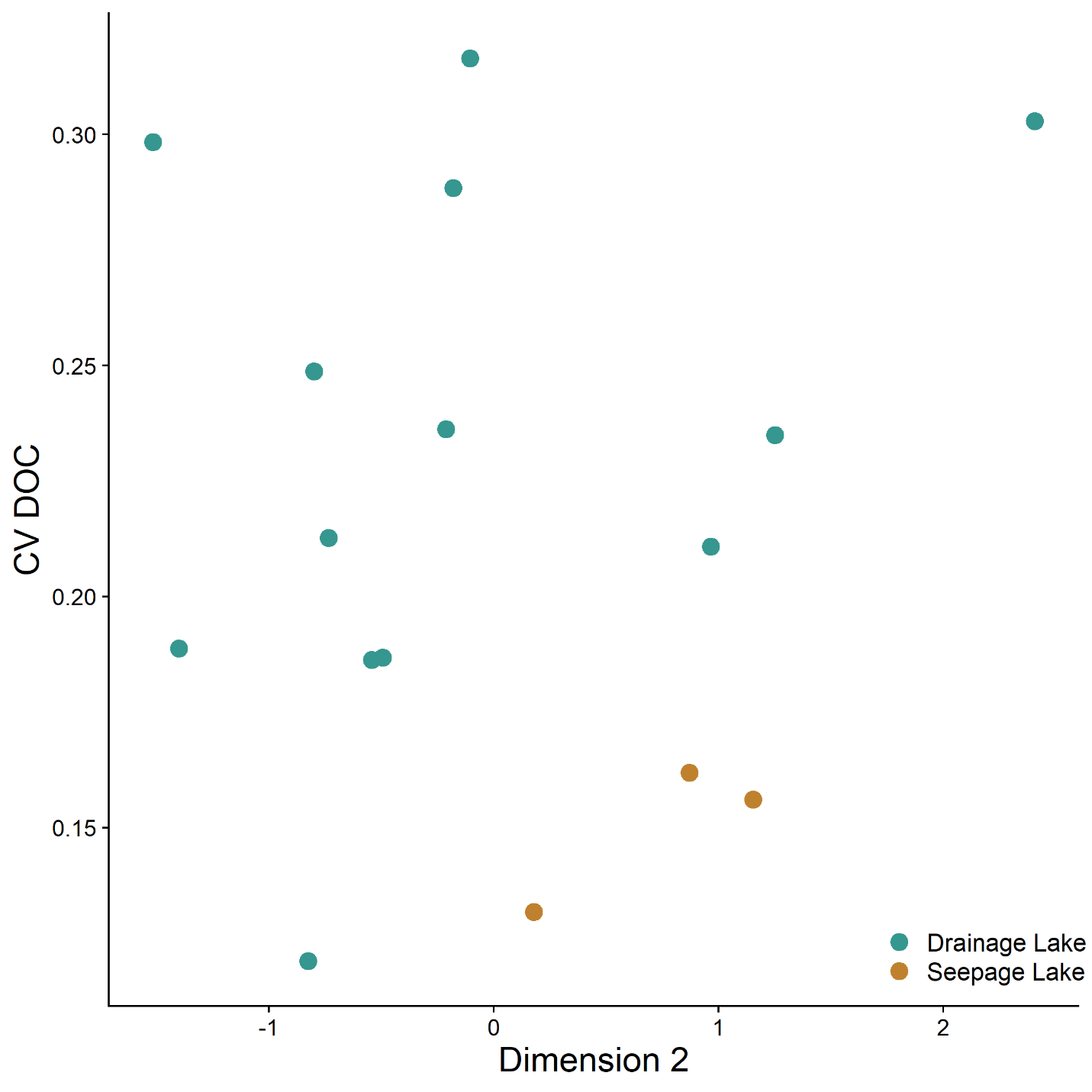
**

**Supplementary Figure 3.** Relationship between the second axis of the Multiple Factor Analysis (Dimension 2) describing watershed characteristics and the coefficient of variation in dissolved organic carbon (CV DOC). Each point represents the CV in DOC for each lake. Colors denote lake type.
